# Long-term satisfaction of patients after laparoscopic and robotic-assisted hysterectomy

**DOI:** 10.1007/s00404-021-06360-9

**Published:** 2021-12-26

**Authors:** Georgios Gitas, I. Alkatout, L. Proppe, L. Hanker, L. Allahqoli, G. Grimbizis, A. Rody, N. Werner, S. Sommer, S. Baum

**Affiliations:** 1 Department of Obstetrics and Gynecology, University Hospital of Schleswig Holstein, Campus Luebeck, Ratzeburger Allee 160, Haus A, 23538 Luebeck, Germany; 2 Department of Obstetrics and Gynecology, University Hospital of Schleswig Holstein, Campus Kiel, Kiel, Germany; 3 School of Public Health, Iran University of Medical Sciences (IUMS), Tehran, Iran; 4 First Department of Obstetrics and Gynecology, Aristotle University of Thessaloniki, Thessaloniki, Greece

**Keywords:** Da Vinci Xi, Laparoscopic hysterectomy, Questionnaire, Robotic surgery, Patient satisfaction

## Abstract

**Introduction:**

Da-Vinci-Xi is the most recent device used in gynecologic robotic surgery. The aim of the present study was to compare the long-term satisfaction of patients who had undergone conventional laparoscopic hysterectomy or robotic assisted laparoscopic hysterectomy using the Da-Vinci-Xi surgical system.

**Methods:**

All hysterectomies performed at the University Hospital of Luebeck from 2018 to 2019 were reviewed. Postoperative outcomes were compared between women who had undergone total hysterectomy with da Vinci Xi (*n* = 42) or conventional laparoscopy (*n* = 97). Postoperative outcomes included pain, elimination of complaints after surgery, bladder function, sexual function, satisfaction with the cosmetic outcome, positive experiences after robotic surgery, and satisfaction with the surgeon’s preoperative explanation. Obese patients were evaluated separately in a subgroup analysis.

**Results:**

Both groups had similar baseline characteristics and complication rates. Preoperative complaints subsided after surgery in a little more than 90% of patients. No significant differences were noted between groups in this regard (*p* = 0.262), or with reference to postoperative pain after one week (*p* = 0.866) and one month (*p* = 0.580), stress incontinence (*p* = 0.343), sexual function (*p* = 0.766) and the cosmetic outcome of the abdominal incisions (*p* = 0.273). The majority of patients who had undergone robotic surgery (96.8%) would be willing to undergo the procedure again if necessary. The subgroup analysis of obese patients revealed no significant differences.

**Conclusion:**

The Da-Vinci-Xi device did not improve the long-term surgical satisfaction of normal-weight or obese patients who underwent hysterectomy compared with patients who underwent conventional laparoscopy performed by experienced laparoscopic surgeons.

## Introduction

Indications for robotic surgery have expanded considerably since FDA approval of the da-Vinci robot in 2005. In the United States, the use of robotic-assisted procedures in general increased from 1.8% in 2012 to 15.1% in 2018 [1]. Robotic-assisted laparoscopic hysterectomy is the most common robotic-assisted operation in gynecology, and the second most commonly performed surgery after cesarean section [2, 3]. Da-Vinci-Xi, the most recent device in robotic surgery, is an advancement of the da Vinci robot in terms of form and functionality, including its targeting ability and the option of moving the robotic camera from port to port [4].

Extreme obesity, expressed as a body mass index (BMI) ≥ 40 kg/m^2^, is emerging as a major public health problem; its prevalence has increased more than fourfold since the mid-1980s [5]. Obesity appears to be associated with a lower risk of morbidity in patients undergoing robotic-assisted gynecological surgery [6].

Despite the above-mentioned advantages, studies comparing the benefits and limitations of robotic-assisted hysterectomy with traditional laparoscopic hysterectomy remain inconclusive [7, 8]. The existing studies show that patients who underwent surgery with the da-Vinci device had favorable outcomes in terms of quality of life and were generally pleased with their decision to undergo robotic-assisted surgery [9]. Studies concerning the satisfaction of patients with the robotic approach after 6 months or longer are still missing.

The aim of the present study was to compare long-term satisfaction in women who had undergone conventional laparoscopic surgery versus those who had undergone robotic-assisted total hysterectomy with Da-Vinci-Xi. The addressed aspects of satisfaction included sexual intercourse, postoperative pain, convalescence, incontinence, and cosmetic outcomes or scars. The surgical outcome of robotic hysterectomy in obese patients was analyzed separately.

## Materials and method

A cohort study was carried out at the department of obstetrics and gynecology, University of Luebeck, from 2018 to 2019. Two-hundred women who had undergone hysterectomy by conventional laparoscopy were compared with one-hundred women who had undergone hysterectomy with the Da-Vinci-Xi device. The selections of the patients were based on the patient’s wish to undergo robotic surgery or not and on our operational capacity'; thus it was limited for robotic surgery only once a week. The inclusion criteria were women who had undergone robot-assisted hysterectomy with Da-Vinci-Xi for benign indication (bleeding disorders, growth of uterus myomas, dyspareunia or abdominal pain) or early endometrial cancer. Patients with a tumor stage higher than FIGO I or with an additional operative procedure to hysterectomy, such as a prolapse operation or lymphadenectomy, were excluded from the study. The study was approved by the local ethics committee and our clinical protocols were in accordance with the German guidelines [10–13].

All surgeries were performed by three surgeons who had completed a course in robotic-assisted gynecologic surgery, and were also experienced in laparoscopic surgery (more than 500 laparoscopies). On the other hand, they had no experience of performing robotic surgery in humans prior to the study period. The surgeons’ learning curve was completed in the middle of the study.

The preoperative explanation included a written description of the procedure and its risks. Patients were required to give their written consent. In the laparoscopic group, the abdominal cavity was accessed through the umbilicus and three trocars (5 and 10 mm) were inserted at the lower quadrant of the abdomen. In the robotic group, the abdominal cavity was accessed through the umbilicus and an 8-mm trocar was inserted here. The remaining four trocars (three robotic 8-mm trocars and one 10-mm trocar for the assistant) were placed in a straight line at the level of the umbilicus.

Baseline characteristics (Table 1) were obtained from the patients’ medical records. Postoperative outcomes included pain, elimination of complaints after surgery, bladder function, sexual function, satisfaction with the cosmetic outcome, positive experiences after robotic surgery, and satisfaction with the surgeon’s preoperative explanation.

**Table 1:** Demographic data of patients who had undergone surgery by the laparoscopic or robotic approach

	Group I (*n* = 97)	Group II (*n* = 42)	Total	*p*
Age (years)	54.874 ± 13.196	56.450 ± 13.185	55.400 ± 13.166	**0.495**^**†**^
BMI (kg/m^2^)	29.596 ± 8.21	32.639 ± 8.676	30.522 ± 8.441	**0.026**^**†**^
Obesity				**0.233**^**††**^
25–29.9	30 (30.9%)	11 (26.2%)	41 (29.5%)	
30–34.9	21 (21.6%)	10 (23.8%)	31 (22.3%)	
39–39.9	8 (8.2%)	7 (16.7%)	15 (10.8%)	
> 40 kg/m^2^	9 (9.3%)	7 (16.7%)	16 (11.5%)	
Parity, *n*				**0.403**^**†††**^
0	28 (30.1%)	9 (22.0%)	37 (27.6%)	
1–3	62 (66.7%)	29 (70.7%)	91 (67.9%)	
> 3	3 (3.2%)	3 (7.3%)	6 (4.5%)	
Menopause	53 (76.8%)	25 (78.1%)	78 (77.2%)	**0.884**^**††**^
Diabetes	12 (12.4%)	4 (9.5%)	16 (11.5%)	**0.776**^**†††**^
ASA physical status classification system				**1.000**^**†††**^
1	15 (15.5%)	3 (7.3%)	18 (13.0%)	
2	58 (59.8%)	26 (63.4%)	84 (60.9%)	
3	23 (23.7%)	12 (29.3%)	35 (25.4%)	
4	1 (1.0%)	0 (0%)	1 (0.7%)	
Previous abdominal surgery				**0.444**^**†††**^
0	52 (54.2%)	18 (42.9%)	70 (50.7%)	
1	19 (19.8%)	13 (31.0%)	32 (23.2%)	
2	14 (14.6%)	7 (16.7%)	21 (15.2%)	
3	5 (5.2%)	2 (4.8%)	7 (5.1%)	
4	4 (4.2%)	1 (2.4%)	5 (3.6%)	
Weight of the uterus (g)	284.17 ± 251.85	177.00 ± 102.158	251.77 ± 221.941	**0.229**^**†**^
Blood loss (g/dl)	1.611 ± 1.825	1.419 ± 0.86	1.52 ± 1.42	**0.512**^**†**^
Intraoperative complications	1 (1.0%)	1 (2.4%)	2 (1.4%)	**0.515**^**†††**^
Clavien-Dindo Grade IIIa	7 (7.2%)	3 (7.1%)	10 (7.2%)	**0.647**^**†††**^
Postoperative complications	3 (3.1%)	1 (2.4%)	4 (2.8%)	**0.307**^**†††**^
Reoperation needed	8 (8.2%)	3 (7.1%)	11 (7.9%)	**0.563**^**†††**^

Continuous values are presented as means ± SD, and categorical variables are shown in numbers (%)

Group I: Conventional laparoscopy, Group II: Robotic-assisted

*y* year, *n* number, *g* gram, *BMI* body mass index, *ASA* American Society of Anesthesiologists, *g/dl* grams per decilitre

^†^Mann–Whitney *U* test

^††^*χ*^2^ test

^†††^Fisher's exact test

*p*-value more than 0.05 is statistically not significant

Pain was measured 1 week and 1 month after surgery. In order to avoid bias resulting from different cognitive levels, we used a numeric rating scale (NRS) for pain (0 = no pain, 10 = worst pain imaginable) as recommended by the Initiative on Methods, Measurement and Pain Assessment in Clinical Trials (IMMPACT) [14]. Bladder function parameters included change of function after surgery and incontinence during physical activity. Sexual function consisted of four questions: sensation during intercourse after surgery, altered frequency of intercourse, reasons for the altered frequency of intercourse, and change in satisfaction during intercourse.

We used a questionnaire designed by the study group. The questions, which were reviewed by the consultant surgeons and validated for patient use, were based on two validated questionnaires: the Female Sexual Function Index (FSFI) [15, 16] and the health-related quality of life questionnaire by the EuroQol Group (EQ-5D) [17, 18]. Patients were asked to evaluate their long-term satisfaction with the procedure. Patients with partially filled questionnaires were excluded from the study.

A subgroup analysis was performed in patients with early endometrial cancer, benign disease, and obesity. According to the international classification, obesity is defined as a BMI ≥ 30 kg/m^2^, and extreme obesity as a BMI ≥ 40 kg/m^2^ [19].

All data were entered in the statistical software program IBM SPSS Statistics for Windows, version 21.0 (IBM Corp. 2012. Armonk, NY: IBM Corp.). Qualitative variables were described by frequency (percentage) and compared between groups with the Chi-square test or Fisher's exact test as appropriate. Normal distribution of data was assessed using a one-sample Kolmogorov–Smirnov test. The Mann–Whitney *U* test was used to compare differences between two groups.

## Results

One hundred and thirty-nine patients returned the completed questionnaire, which corresponds to a response rate of 46.3%. The patients’ mean age (± SD) was 55.4 ± 13.1 years; the youngest woman was 36 years old and the oldest 79 years. Approximately 77% of patients were postmenopausal. Thirty-one of them underwent surgery for early endometrial cancer. The mean weight of the uterus was 251.8 g. The most common benign indications for surgery were bleeding disorders or uterine myomas (91.3%).

Conventional laparoscopic hysterectomy was performed in 97 patients (Group I) and robot-assisted hysterectomy with Da-Vinci-Xi in 42 patients (Group II). The indications for hysterectomy were similar in both groups (*p* = 0.641). Blood loss, intra- and postoperative injuries, and reoperation rates were similar in both groups. Patient characteristics were also similar and are summarized in Table 1.

**Fig. 1:**
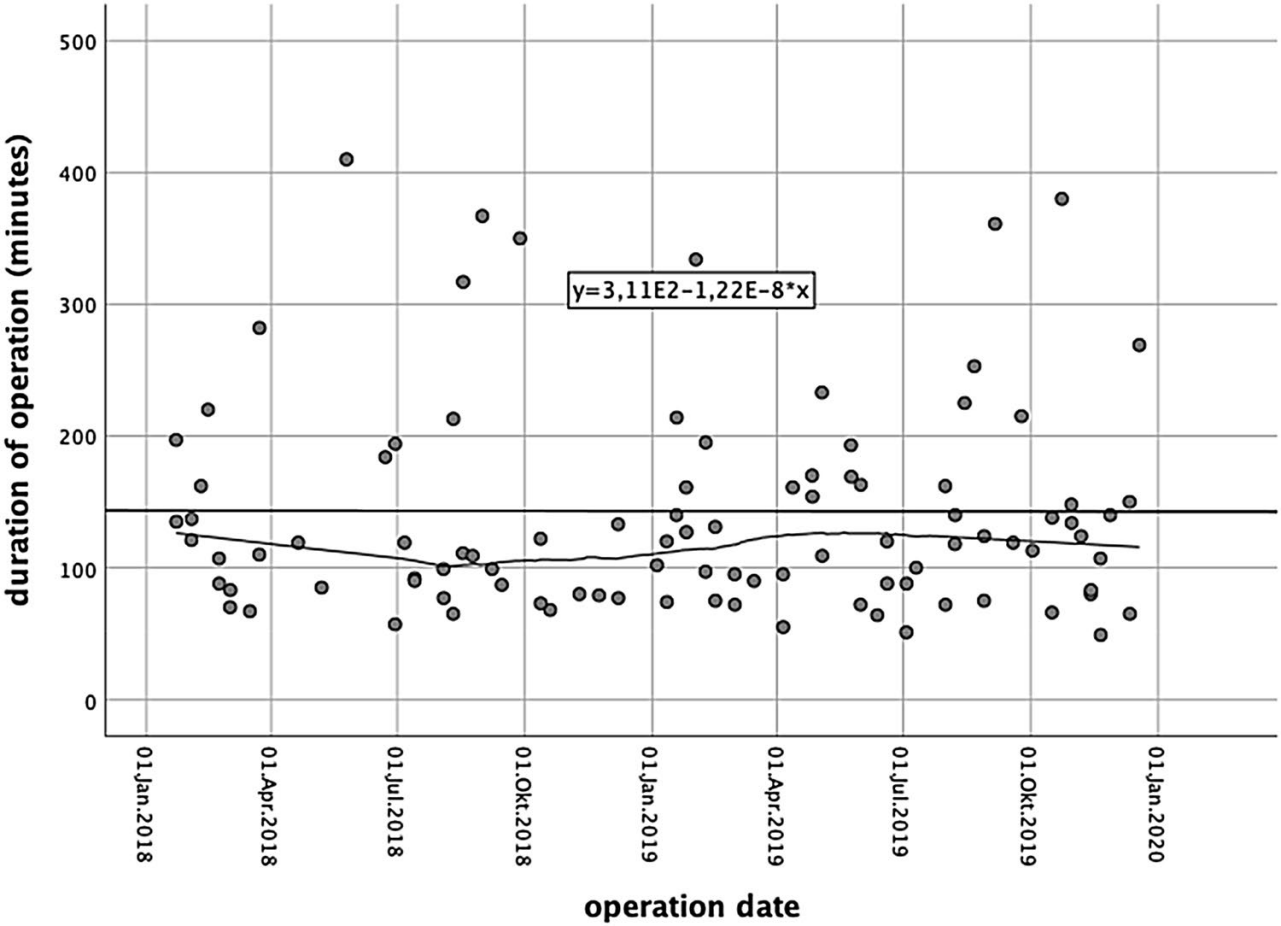
Time taken by our team to perform robotic-assisted surgery (learning curve)

With regard to the surgeons’ learning curve (hysterectomies without follow-up were also included: 300 cases), the last 50 robotic surgeries were slightly shorter (141.54 ± 79.4 vs. 144.38 ± 86.57 min) than the first 50 surgeries, but the difference was not statistically significant (*p* = 0.945) (Fig. 1). No significant differences were noted between groups with regard to postoperative pain after 1 week or 1 month, or any of the above-mentioned outcomes (Table 2). Preoperative complaints subsided after surgery in a little more than 90% of patients.

**Table 2:**
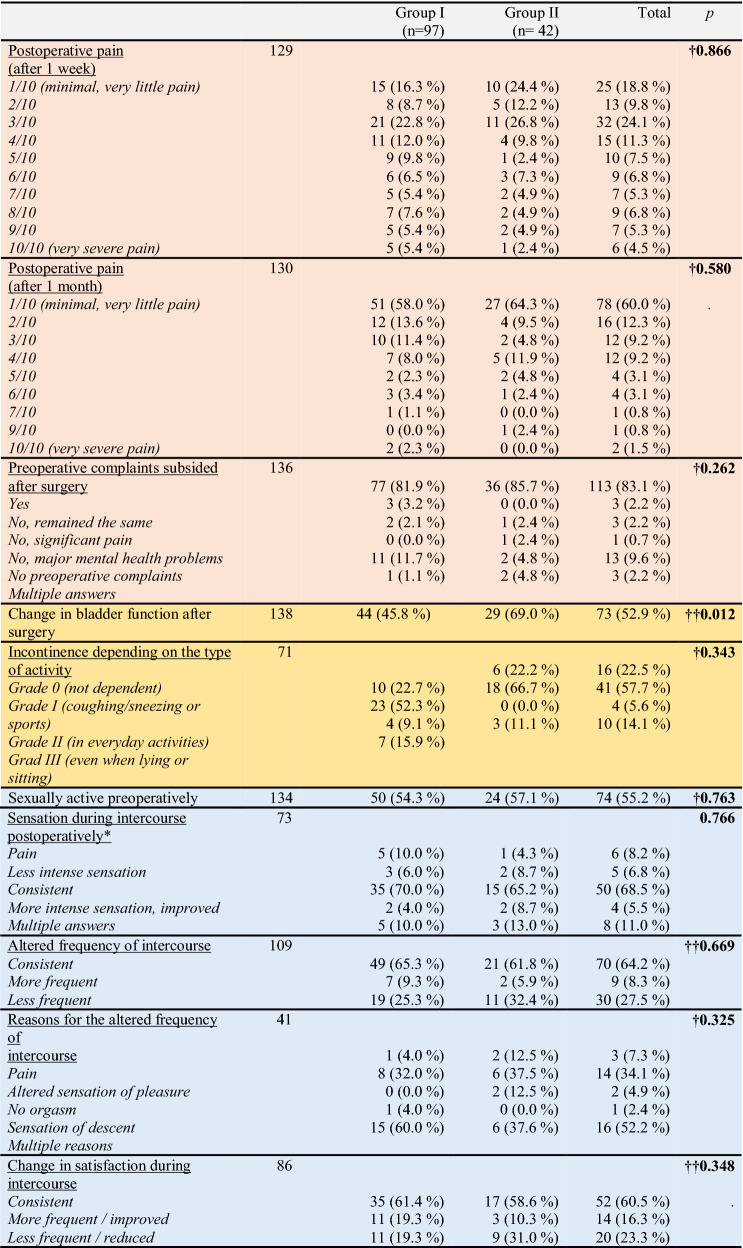

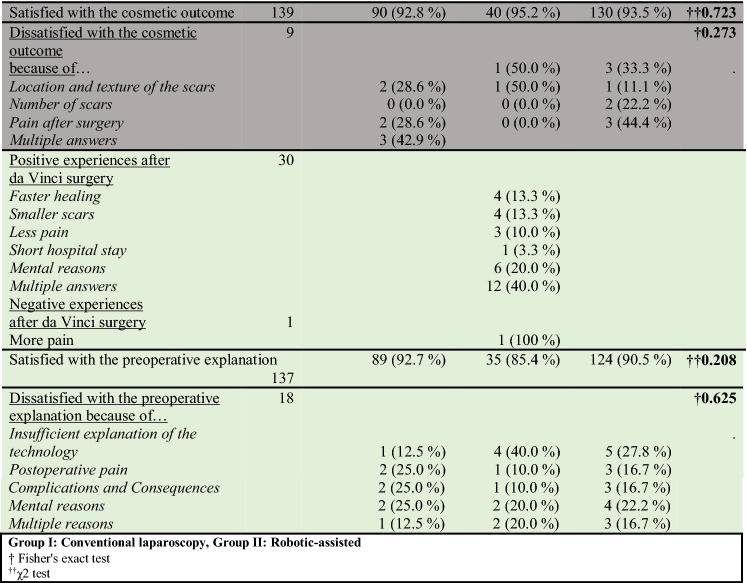
Long-term postoperative satisfaction based on the questionnaire

Color categories: pink: pain, yellow: continence, blue: sexual function, gray: cosmetic, green: satisfaction and preoperative explanation

Group I: Conventional laparoscopy, Group II: Robotic-assisted

^†^Fisher's exact test

^††^*χ*^2^ test

Significantly more patients in the robotic surgery group experienced a change in bladder function postoperatively (69.0 vs. 45.8%). However, stress incontinence did not differ significantly between the two groups (*p* = 0.342). The rate of postoperative stress incontinence (Grade I–III) was 24.6% in the laparoscopic group and 18.0% in the robotic group. Data concerning grades of stress incontinence are shown in Table 2.

Only 55.2% of the patients were sexually active preoperatively. Approximately two-thirds of patients in both groups had consistent sexual function postoperatively. Satisfaction during intercourse was improved in 16.3%, and reduced in 23.3%. The most common reason for the altered frequency of intercourse was a change in the sensation of pleasure (34.1%), followed by pain during intercourse (7.3%). There was no significant difference in this regard between the laparoscopic and robotic group (Table 2).

In all, 93.5% of patients (*n* = 139) were satisfied with the cosmetic outcome of the abdominal incision. The percentages in the respective groups were 92.8% for laparoscopic surgery and 95.2% for robotic surgery (*p* = 0.723). Detailed interviews with patients revealed that only four patients in each group were dissatisfied with the numbers and locations of scars. The majority of patients who had undergone robotic surgery (96.8%, *n* = 30/31) would undergo the procedure again if necessary; the various reasons are shown in Table 2.

Furthermore, 90.5% of all patients were satisfied with the preoperative explanation of the operation. However, 40% (4/10) of the dissatisfied patients in the robotic group were poorly informed about the technology.

The subgroup analysis of patients who had undergone surgery only for early endometrial cancer or benign indications revealed no significant differences between the two subgroups. The results are shown in Table 3. Furthermore, we found no significant difference in patients with a BMI > 30 kg/m^2^ or a BMI > 40 kg/m^2^ in regard of postoperative outcomes and satisfaction.

**Table 3:**
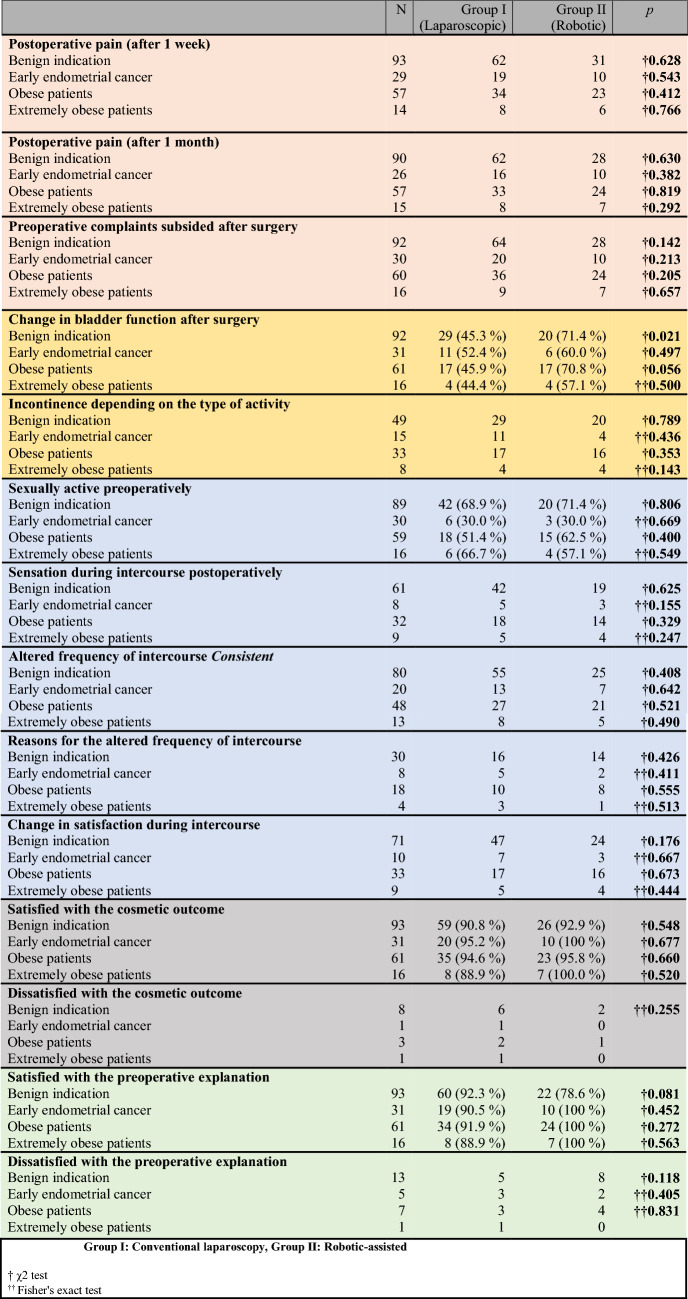
Subgroup analysis of long-term postoperative satisfaction with reference to the indications for surgery (benign vs. early endometrial cancer), BMI ≥ 30 kg/m^2^ (obesity), and BMI ≥ 40 kg/m^2^ (extreme obesity)

Color categories: pink: pain, yellow: continence, blue: sexual function, gray: cosmetic outcome, green: preoperative explanation

Group I: Conventional laparoscopy, Group II: Robotic-assisted

^†^*χ*^2^ test

^††^Fisher's exact test

## Discussion

In the present study, we compared the long-term satisfaction of patients who had undergone hysterectomy using total laparoscopic surgery versus robotic surgery with Da-Vinci-Xi. The robotic approach yielded similar results as the laparoscopic procedure performed by well-trained surgeons in regard of postoperative satisfaction, sexual function, incontinence, cosmetic results, and pain. The majority of patients who had undergone robotic surgery were willing to undergo the procedure again if necessary.

Robotic-assisted laparoscopy is considered potentially superior to conventional laparoscopy in regard of postoperative pain. Studies assessing short-term perioperative parameters have reported that robotic-assisted laparoscopy is not associated with larger narcotic doses or greater postoperative pain compared to conventional laparoscopy [20, 21]. We used a numeric rating scale from 0 to 10 to assess pain and avoid bias resulting from different cognitive levels. We observed no significant difference among the two groups.

Quality of life at 6 months post-surgery was reported to be significantly better in patients who had undergone total laparoscopic hysterectomy compared to those who had undergone total abdominal hysterectomy [22]. However, patient satisfaction and quality of life have been scarcely investigated after robotic surgery. Two trials analyzed quality of life in patients who underwent conventional total laparoscopy hysterectomy or robotic-assisted hysterectomy [23, 24]. In 2013 Paraiso et al. randomized 62 women, followed them for 6 months after robotic and conventional laparoscopic surgery, and registered no significant difference in regard of quality of life [24]. In 2012, Sarlos et al. evaluated self-care, activities of daily living, pain, mobility, discomfort, depression, and anxiety in 96 patients after hysterectomy, and reported a significantly greater change in the preoperative to postoperative quality-of-life index in the robotic group compared to the laparoscopic group. However, the authors observed no difference in long-term outcomes [23]. Despite the ongoing development of robotic-assisted surgery, we registered similar results many years later.

The majority of our patients (90.5%) were satisfied with the information they received prior to the operation. Satisfaction rates were slightly lower in the robotic group than in the laparoscopic group, but the difference was not statistically significant (85.4 vs. 92.7%). However, the majority of dissatisfied patients lacked information about the technology, which is prone to rapid development and may aggravate the patients’ anxiety. This issue should not be underestimated because thorough preoperative enlightenment enhances the patient’s active participation in care and may well contribute to a rise in overall satisfaction [25].

More than a half of our patients were sexually active preoperatively. A significant number of them had trouble resuming sexual intercourse at least 6 months postoperatively (30% of the laparoscopic, and 34.8% of the robotic group). Both groups had similar data in this regard. Our search of the published literature yielded no similar studies. In 2014, De La Cruz et al. analyzed postoperative sexual function and vaginal length in 38 patients who underwent total vaginal hysterectomy and in 46 who underwent robotic hysterectomy [26]. The authors observed no difference in sexual function between the two groups, but noted a greater reduction of vaginal length in the vaginal hysterectomy group. In 2016, Ercan et al. compared the vaginal and laparoscopic approach, and reported similar data [27].

Ercan et al. concluded that the absence of visible abdominal scars is probably associated with positive effects on the patients’ self-esteem and quality of life [27]. We observed no significant difference between our groups concerning cosmetic results following the trocar incisions. More numerous trocars in a straight line at the level of the umbilicus were placed in the robotic group (Da-Vinci-Xi) in contrast to the laparoscopic group. Patient satisfaction with the cosmetic outcome was more than 92% in both groups. Elessawy et al. reported that 20% of patients were dissatisfied with the abdominal incision after total hysterectomy in the robotic group and only 2.7% in the laparoscopic group [28]. The authors attributed the difference to the rigidity of robotic trocars compared to the disposable trocar used in laparoscopy. However, a direct comparison of the latter study with ours is hindered by the fact that patients were contacted earlier (three weeks postoperatively), and the authors did not use the Da-Vinci-Xi system (incisions in a straight line). The development of a small single-incision port for robotic surgery may enhance satisfaction with the cosmetic outcome.

Significantly more patients experienced a change in postoperative bladder function in the robotic surgery group (69.0 vs. 45.8%). Possibly the advantages of robotic surgery (3D techniques, finer tissue dissection) reduced the incidence of postoperative pelvic floor dysfunction by avoiding injury to local nerves and other pelvic floor structures. However, we focused on postoperative stress incontinence. With a prevalence of 14.3%, stress incontinence is the most common complication in the lower urinary tract after hysterectomy [29]. On the other hand, a recent meta-analysis showed that hysterectomy for benign gynecological conditions does not increase the risk of adverse urinary symptoms [30]. We registered no statistically significant difference between groups concerning postoperative stress incontinence. However, postoperative stress incontinence rates were relatively high and slightly higher in the laparoscopic group (24.6%) than in the robotic group (18.0%).

The foremost limitation of the present study is that it was conducted at a single unit. The interval between surgery and the patients’ response to the questionnaire may be viewed as a strength as well as a limitation of the study. Presenting the questionnaire to the patients after 6 months post-surgery may have led to bias. However, the aim of the study was to estimate long-term outcomes. A strength of the study was that we evaluated the most recent version of the robotic device, Da-Vinci-Xi, and also compared it directly with conventional laparoscopy. Furthermore, we compared the surgical outcome of laparoscopic approach with the robotic approach by trained laparoscopic surgeons without experience on robotic surgery, which according to our opinion, is an option and does not make our groups inhomogeneous but unique; thus most of the other institutions had already experienced robotic surgery before using Da-Vinci-Xi system. Our analysis revealed no significant difference in obese or extremely obese patients in regard of postoperative satisfaction. To the best of our knowledge, quality of life in obese patients after robotic hysterectomy has not been addressed in any published study.

However, we need further data on the use of robotic surgery in gynecology, especially with regard to long-term outcomes such as returning to activities of daily living. Further development of robotic surgery such as single port technique with smaller and flexible instruments and intraoperative high-quality navigation with imaging systems could open fascinating new avenues for improved minimally invasive surgery [31]. During this period, accumulating evidence has demonstrated the advantages and feasibility of vaginal natural orifice transluminal endoscopic surgery, which unfortunately is not suitable for complex gynecological operations [32, 33]. In an attempt to overcome the shortcomings of the vaginal access, robotic surgery might be the solution.

## Conclusion

Our data suggest that conventional total laparoscopic hysterectomy, performed by trained laparoscopic surgeons, is a safe procedure for a benign indication or for early endometrial cancer, and equivalent to robotic-assisted surgery in regard of postoperative patient satisfaction, sexual function, incontinence, cosmetic results, and pain. The majority of patients were willing to undergo robotic surgery again if necessary. The Xi-robot and the total laparoscopic procedure for hysterectomy yielded similar outcomes in obese patients, and no difference in patient satisfaction. Ongoing application and evaluation of the technology may be expected to optimize its function. The use of robotic surgery for a variety of gynecological diseases is yet to be clarified. Prospective multicenter randomized studies will be needed to determine the exact role of robotic surgery in gynecological surgery.

## Data Availability

The datasets used and analyzed in the current study are available from the corresponding author on reasonable request.
